# Estimating an intervention’s effects on health care disparities within and between physician organizations: decomposing the effects of health system affiliation

**DOI:** 10.1007/s10742-025-00350-z

**Published:** 2025-06-14

**Authors:** Maria DeYoreo, Denis Agniel, Shiyuan Zhang, José J. Escarce, Justin W. Timbie

**Affiliations:** 1 RAND, 1776 Main Street, Santa Monica, CA 90401 USA; 2 RAND, 1200 S Hayes St, Arlington, VA 22202 USA; 3 Department of General Internal Medicine, UCLA, 911 Broxton Plaza, Los Angeles, CA 90095 USA

**Keywords:** Health care disparities, Hierarchical regression models, Quality of care, Statistical modeling

## Abstract

Vertically integrated health systems are expanding across the U.S., but there is no evidence that system-affiliated providers are better able to reduce socioeconomic disparities than unaffiliated providers. This paper develops and interprets statistical models for estimating the longitudinal effects of an exposure, health system affiliation, on within- and between-group disparities. Mixed effect difference-in-differences regression models were used to estimate the effect of physician organization (PO) health system affiliation on socioeconomic disparities in diabetes medication adherence for fee-for-service Medicare beneficiaries from 2013 to 2019, decomposing the effect of affiliation on disparities into within- and between-PO socioeconomic disparities. We find that after becoming affiliated, adherence for dually-eligible beneficiaries worsened relative to non dually-eligible beneficiaries within POs, but between-PO disparities were reduced. Simulation studies were used to evaluate the methods, and showed the importance of centering dual-eligibility status and its interaction with affiliation status at the PO-year level and including the PO-year level means as predictors to decompose effects of affiliation into within and between-PO effects.

## Introduction

Vertically integrated health systems, which are health care organizations that unite hospitals and physician organizations (POs) under common ownership (O'Malley et al. 2011) are expanding across the U.S, with the percentage of physicians that work in hospital-owned practices increasing from 26 to 52% from 2012 to 2022 (American Medical Association 2023). There is limited evidence that system-affiliated POs provide higher quality care, with findings of positive impacts on structural measures but not necessarily on quality or outcome measures (Hwang et al. 2013; Machta et al. 2019).

While the effect of affiliation on quality overall is still unclear, health systems may be well-positioned to address socioeconomic and racial and ethnic disparities, which are large and persistent, with no meaningful improvement between 2000 and 2021 in any of the socioeconomic-related disparities that existed in 2000 among those examined in an AHRQ report (Agency for Healthcare Research and Quality 2023). POs that affiliate with health systems may benefit from greater access to practice infrastructure such as advanced EHR systems and enhanced access to specialists and other staff (Rudin et al. 2020), such as community health workers (Jack et al. 2017), that can help identify care gaps, improve coordination, and enhance patient experiences in ways that reduce disparities for high need and minoritized patients (Knowles et al. 2023). On the other hand, health system affiliation raises concerns about health equity if physicians experience reduced autonomy over clinic operations or if health systems use new appointment scheduling processes or policies that create barriers accessing services or the amount of time during visits, which could widen disparities for high-need patients (Jain 2024).

In this study, we examine the effects of health system affiliation on disparities (e.g., differences in quality measure scores between a minoritized or disadvantaged group and a majority or advantaged group), using diabetes medication adherence rates as an illustrative example. One SES disparity of interest is between beneficiaries who are dually eligible (DE) for Medicare and Medicaid and those who are not. Such an assessment is timely given the rapid growth of health systems and existing health disparities. To gain a complete understanding of the impact of health system affiliation on disparities, we aim to decompose the overall effect of affiliation on disparities both within and between physician organizations (POs). The within-PO effect of affiliation on disparities represents the average effect of becoming affiliated on disparities within a PO. Between-PO disparities describe differences in average outcomes by composition of the PO, and indicate whether a disadvantaged group is concentrated in lower performing POs. The effect of affiliation on between-PO disparities thus describes how affiliation might narrow differences in quality between POs that disproportionately serve duals and those that serve few duals.

Both types of disparities, within and between, are important from a policy perspective, but suggest different levers to target to reduce disparities. If the effect of affiliation is to worsen within-PO disparities, this might suggest discouraging health system expansion or providing incentives to health systems to reduce disparities and improve quality for the disadvantaged group. On the other hand, if the effect is positive, this indicates that health systems provide resources or incentives for their POs to reduce disparities. If affiliation worsens between-PO disparities, this indicates that affiliation might be differentially benefiting high-SES POs and/or differentially harming low-SES POs, and disadvantaged beneficiaries may need more access to better performing providers within their health system.

There is a large literature on disentangling within and between-unit effects in multilevel or hierarchical data structures (Raudenbush and Bryk 2002; Dieleman and Templin 2014; Bell and Jones 2015; Bell et al. 2019; Hamaker and Muthen 2020). In multilevel modeling, typical data structures can be cross-sectional, with individuals nested within groups, or longitudinal, such as repeated measures data with occasions nested within individuals or units. In the cross-sectional setting, it is important to separate the within-unit slope from the between-unit slope because there may be different relationships between the predictor and the outcome at different levels. The literature (Bell et al. 2019; Hamaker and Muthen 2020) explains how random effects models with random unit-level intercepts can be used to estimate both level 1 and level 2 relationships between a predictor and an outcome by centering the individual time-varying predictor at the individual-level mean and including the unit-level (time-invariant) mean as a predictor. In the longitudinal data setting, level 1 “within-unit” relationships describe the effect of a unit-level variable changing over time (e.g., the effect of an individual’s education level changing over time), and level 2 “between-unit” relationships describe the relationship between the average, time-invariant, unit-level characteristic and the outcome (e.g., the effect of having more or less education in general, across all time points).

The data structure for the analysis at hand is comparative longitudinal or repeated cross-sectional, with beneficiaries nested within PO-years, and PO-years nested within POs (e.g., there are repeated measures on POs over multiple years, but the beneficiaries in each PO are not the same across years). Fairbrother (2014) focuses on multilevel modeling techniques in the comparative longitudinal setting, and emphasizes the importance of distinguishing cross-sectional and longitudinal relationships, analogously to distinguishing between- and within-unit relationships.

The literature focusing on disentangling within- and between-group effects provides a framework for estimating within- and between-PO disparities, or within- and between-PO effects of an individual characteristic (e.g., an indicator of DE) on a quality measure outcome. Published methods would be directly applicable to our study if we were interested in the longitudinal and cross-sectional effects of affiliation, a PO-year level variable, on health care quality outcomes. However, we are interested in the longitudinal effects of affiliation on within-PO disparities, and in the effects of affiliation on between-PO disparities. While the literature provides plenty of guidance on using multi-level modeling to estimate the within- and between-cluster effects of an intervention, it does not provide guidance on how to estimate effects of interventions on within and between-cluster disparities. These quantities are both captured by interaction terms between affiliation status and an individual-level or PO-level characteristic (e.g., DE indicator at the individual-level or percent DE at the PO-level). In this paper, we expand on the multi-level modeling approaches provided in the literature to estimate longitudinal and cross-sectional relationships between an outcome and an interaction between a group-year level variable (PO-year level in our context) and an individual-level variable. This is an important contribution because of the growing interest in interventions to reduce health disparities, and because individuals are often naturally clustered or grouped into units such as hospitals, practices, or geographic areas.

The rest of this manuscript proceeds as follows. In Sect. 2, we present the proposed mixed effects model for capturing the effects of affiliation on within- and between-PO disparities and provide interpretations of each model coefficient. In Sect. 3, we conduct simulation studies to compare the performance of the proposed model to alternatives, and we apply the model to analyze the impact of health system affiliation on disparities in diabetes medication adherence. Section 4 concludes with a Discussion.

## Methods

### Statistical model

Let $y_{ijt}$ be an outcome variable for individual $i$, such as a quality measure score, in group (PO) $j$, at time (year) $t$, where $i = 1,2, \ldots ,n_{jt}$, $j = 1,2, \ldots ,g_{t}$, and $t = 1,2, \ldots ,T$. Let $x_{ijt}$ be an individual-level characteristic that defines the disparity of interest, such as an indicator of DE in our application. Let $z_{jt}$ be a group-time level characteristic, such as affiliation status of PO $j$ in year $t$. To facilitate notation, define $I_{ijt} = x_{ijt} z_{jt}$, an interaction between the beneficiary-level characteristic and the group-time level characteristic. We use the following multi-level linear model to understand the effects of affiliation on disparities.

1 $$ \begin{aligned} y_{ijt} = & \beta_{0} + \beta_{w1} \left( {x_{ijt} - \overline{x}_{jt} } \right) + \beta_{b1} \overline{x}_{jt} + \beta_{w2} \left( {I_{ijt} - \overline{I}_{jt} } \right) + \beta_{b2} \overline{I}_{jt} + \beta_{w3} \left( {z_{jt} - \overline{z}_{j} } \right) \\ & + \beta_{b3} \overline{z}_{j} + \delta_{t} + u_{j} + u_{jt} + e_{ijt} . \\ \end{aligned} $$

here $\delta_{t}$ are year dummies, with $\delta_{1} = 0$, $u_{j} \sim N\left( {0,\sigma_{u1}^{2} } \right)$ are random intercepts for PO, $u_{jt} \sim N\left( {0,\sigma_{u2}^{2} } \right)$ are random intercepts for PO-year, and $e_{ijt} \sim N\left( {0,\sigma_{e}^{2} } \right)$ is an error term. Note that the data may not require both PO-year and PO random intercepts, and PO-year random effects should not be included if the sample size within PO-years is small. For completeness, we include both in our presentation of the model. We refer to this model as $M_{wb}$ since it fully captures the within- and between-effects of affiliation on quality and disparities.

As we will show in Sect. 2.2, the term $\beta_{w1}$ captures the within-PO effect of dual-eligibility on the quality outcome (e.g., within-PO disparities in unaffiliated POs) and $\beta_{b1}$ captures the between-PO effect of dual-eligibility on the quality outcome (e.g., between-PO disparities), which is the difference in average outcomes for high versus low percent dual POs. This follows directly from the literature on disentangling within- and between-group effects. Although not of key interest here, $\beta_{w3}$ captures the effect on the quality outcome for non-duals in 0% dual POs of affiliation changes over time within each PO, and $\beta_{b3}$ is the effect on enduring cross-PO differences in affiliation status (e.g., cross-sectional relationships between affiliation and quality).

The quantities captured by this model that are not represented in other literature are the within-PO effects of affiliation on disparities, which indicates how affiliation changes the within-PO gap between dual-eligible and non-dual beneficiaries, estimated by $\beta_{w2}$, and the effect of affiliation on between-PO disparities, estimated by $\beta_{b2}$. This model therefore combines the within- and between-group random effects model for clustered or grouped data with the model for distinguishing longitudinal and cross-sectional relationships from comparative longitudinal data. It also adds terms to capture the effect of a group-time level variable on the within-group effects of an individual-level variable (e.g., the effect of affiliation status on within-PO disparities in quality), and effect of a group-time level variable on between-group associations between quality and a group-level characteristic (e.g., the effect of affiliation status on between-PO disparities in quality).

### Effects of affiliation on disparities

We are interested in four main quantities: the pre-affiliation within-PO disparity $\tau_{w}$, the pre-affiliation between-PO disparity $\tau_{b}$, the effect of affiliation on the within-PO disparity $\Delta_{w}$, and the effect of affiliation on the between-PO disparity $\Delta_{b}$. We define each quantity and show what this corresponds to in (1).

The within-PO disparity between duals ($X_{ijt} = 1$) and non-duals ($X_{ijt} = 0$) for an unaffiliated PO ($Z_{jt} = 0$) with $\overline{X}_{jt} = \overline{x}_{jt}$ and $\overline{Z}_{j} = \overline{z}_{j}$ can be defined as

$$ \begin{aligned} \tau_{w} = & E\left( {Y_{ijt} |X_{ijt} = 1,\overline{X}_{jt} = \overline{x}_{jt} ,Z_{jt} = 0,\overline{Z}_{j} = \overline{z}_{j} } \right) \\ & - E\left( {Y_{ijt} |X_{ijt} = 0,\overline{X}_{jt} = \overline{x}_{jt} ,Z_{jt} = 0,\overline{Z}_{j} = \overline{z}_{j} } \right) \\ = & \beta_{0} + \beta_{w1} \left( {1 - \overline{x}_{jt} } \right) + \beta_{b1} \overline{x}_{jt} + \beta_{w3} \left( { - \overline{z}_{j} } \right) + \beta_{b3} \overline{z}_{j} \\ & - \left( {\beta_{0} + \beta_{w1} \left( { - \overline{x}_{jt} } \right) + \beta_{b1} \overline{x}_{jt} + \beta_{w3} \left( { - \overline{z}_{j} } \right) + \beta_{b3} \overline{z}_{j} } \right) \\ = & \beta_{w1} . \\ \end{aligned} $$

The contextual effect of dual eligibility for unaffiliated POs can be defined as the difference in expected quality for an individual moving from a 100% versus 0% dual PO, holding individual characteristics constant. This takes the form of

$$ \begin{aligned} \tau_{c} = & E\left( {Y_{ijt} |X_{ijt} = x_{ijt} ,\overline{X}_{jt} = 1,Z_{jt} = 0,\overline{Z}_{j} = \overline{z}_{j} } \right) - E\left( {Y_{ijt} |X_{ijt} = x_{ijt} ,\overline{X}_{jt} = 0,Z_{jt} = 0,\overline{Z}_{j} = \overline{z}_{j} } \right) \\ = & \beta_{0} + \beta_{w1} \left( {x_{ijt} - 1} \right) + \beta_{b1} + \beta_{w3} \left( { - \overline{z}_{j} } \right) + \beta_{b3} \overline{z}_{j} - \left( {\beta_{0} + \beta_{w1} x_{ijt} + \beta_{w3} \left( { - \overline{z}_{j} } \right) + \beta_{b3} \overline{z}_{j} } \right) \\ = & \beta_{b1} - \beta_{w1} . \\ \end{aligned} $$

Between-unit effects are defined as the sum of within-unit effects and contextual effects. Therefore, the between-PO disparity for unaffiliated POs is $\tau_{b} = \tau_{w} + \tau_{c} = \beta_{b1}$.

Next consider the effects of affiliation on these disparities. The effect of affiliation on within-PO disparities can be defined as the change in within-PO disparities after affiliation, relative to pre-affiliation. The within-PO disparity after affiliation can be defined as

$$ \begin{aligned} \tau_{w,A} = & E\left( {Y_{ijt} |X_{ijt} = 1,\overline{X}_{jt} = \overline{x}_{jt} ,Z_{jt} = 1,\overline{Z}_{j} = \overline{z}_{j} } \right) - E\left( {Y_{ijt} |X_{ijt} = 0,\overline{X}_{jt} = \overline{x}_{jt} ,Z_{jt} = 1,\overline{Z}_{j} = \overline{z}_{j} } \right) \\ = & \beta_{0} + \beta_{w1} \left( {1 - \overline{x}_{jt} } \right) + \beta_{b1} \overline{x}_{jt} + \beta_{b2} \overline{x}_{jt} + \beta_{w2} \left( {1 - \overline{x}_{jt} } \right) + \beta_{w3} \left( {1 - \overline{z}_{j} } \right) + \beta_{b3} \overline{z}_{j} \\ & - \left( {\beta_{0} + \beta_{w1} \left( { - \overline{x}_{jt} } \right) + \beta_{b1} \overline{x}_{jt} + \beta_{b2} \overline{x}_{jt} + \beta_{w2} \left( { - \overline{x}_{jt} } \right) + \beta_{w3} \left( {1 - \overline{z}_{j} } \right) + \beta_{b3} \overline{z}_{j} } \right) \\ = & \beta_{w1} + \beta_{w2} . \\ \end{aligned} $$

Thus, the effect of affiliation on within-PO disparities is $\Delta_{w} = \tau_{w,A} - \tau_{w} = \beta_{w2}$.

The effect of affiliation on between-PO disparities can be defined as the difference in between-PO disparities among affiliated POs compared to unaffiliated POs. The contextual effects of dual eligibility in affiliated POs can be defined as

$$ \begin{aligned} \tau_{c,A} = & \left( {Y_{ijt} |X_{ijt} = x_{ijt} ,\overline{X}_{jt} = 1,Z_{jt} = 1,\overline{Z}_{j} = \overline{z}_{j} } \right) - E\left( {Y_{ijt} |X_{ijt} = x_{ijt} ,\overline{X}_{jt} = 0,Z_{jt} = 1,\overline{Z}_{j} = \overline{z}_{j} } \right) \\ = & \beta_{0} + \beta_{w1} \left( {x_{ijt} - 1} \right) + \beta_{b1} + \beta_{w2} \left( {x_{ijt} - 1} \right) + \beta_{b2} + \beta_{w3} \left( { - \overline{z}_{j} } \right) + \beta_{b3} \overline{z}_{j} \\ & - \left( {\beta_{0} + \beta_{w1} x_{ijt} + \beta_{w2} x_{ijt} + \beta_{w3} \left( { - \overline{z}_{j} } \right) + \beta_{b3} \overline{z}_{j} } \right) \\ = & \beta_{b1} + \beta_{b2} - \beta_{w1} - \beta_{w2} . \\ \end{aligned} $$

Therefore, the between-PO disparity for affiliated POs after affiliation is $\tau_{b,A} = \tau_{w,A} + \tau_{c,A} = \beta_{b1} + \beta_{b2}$. This implies that the effect of affiliation on between-PO disparities is $\beta_{b2}$.

We also provide the equations for the effects of affiliation on quality of care separately for duals and non-duals in the Appendix.

### Estimating the effect of health system affiliation on disparities in diabetes medication adherence

We used 100% fee-for-service claims data on Medicare beneficiaries from 2013 to 2019 to derive the diabetes medication adherence quality measure for beneficiaries in POs eligible for analysis. POs correspond to Taxpayer Identification Numbers (TINs) and beneficiaries were attributed to the PO from which a beneficiary received a plurality of his or her primary care evaluation and management visits during the year. For beneficiaries without primary care visits in the year, beneficiaries were attributed to the PO that provided a plurality of services to internal medicine sub-specialists. Additional details on the identification of POs, beneficiary attribution to POs, and measurement of health system affiliations can be found in other published works (Timbie et al. 2020).

POs had to have at least two total physicians and one primary care physician in 2013, had to have at least 30 dual and 30 non-dual beneficiaries in at least one year to be eligible for analysis, and had to bill Medicare in each study year. We excluded POs that were always affiliated during our study period (e.g., started out affiliated in 2013), and those with unstable affiliation patterns (e.g., those that both affiliated and de-affiliated during the study period). The sample for this analysis consisted of 1171 POs and over 2.36 million beneficiary-level records.

Covariates used to adjust quality outcomes included beneficiary and PO characteristics likely associated with the outcome but not under control of POs. These included beneficiary-level age, disability status, area-level socio-economic deprivation index, rural–urban commuting area category, race and ethnicity, risk score, and PO-level size (number of beneficiaries and number of physicians), specialty mix, and PO-level aggregated beneficiary characteristics (mean age, mean risk score, mean area-level deprivation index, percent disabled, percent female, percent in each RUCA category, percent in each race and ethnicity category).

## Results

### Simulation studies

We conduct simulation studies to illustrate how other regression models compare to the proposed model in terms of estimating the relationships of interest. If unaware of the discussion about centering in multi-level models, one might consider simply including the individual-level characteristic, PO-year affiliation status, and its interaction with affiliation status to capture the effects of system affiliation on disparities, as reflected in the following model:

2 $$ y_{ijt} = \beta_{0} + \beta_{1} x_{ijt} + \beta_{2} x_{ijt} z_{jt} + \beta_{3} z_{jt} + \delta_{t} + u_{j} + u_{jt} + e_{ijt} . $$

The PO-year and PO effects could be modeled as random effects, or PO and year effects can be treated as fixed effects, and the interpretation of the regression coefficients will differ (Bell et al. 2019; Hamaker and Muthen 2020). When the within-group and between-group effects are not equal, the random effects model without centering will produce a weighted average of the two effects (e.g., $\beta_{1}$ in (2) will be a weighted average of $\beta_{w1}$ and $\beta_{b1}$ in (1)), and these weights are equal to the precision of the estimates. In our applications of interest, there are far more beneficiaries than there are POs, so $\beta_{1}$ will tend towards $\beta_{w1}$. We refer to (2) as $M_{FE}$ and $M_{RE}$ depending on whether fixed or random effects are used. Note that if the PO and PO-year effects are excluded altogether, this model can be used to estimate the total effect of affiliation on disparities.

If using a multi-level model and aware of the importance of centering to identify within-group effects and separate them from between-group effects, it might be clear that the individual-level characteristic $x_{itj}$ needs to be centered at the PO-level to be interpreted as a within-PO effect on quality, and thus one might fit the following model with centering of $x_{ijt}$:

3 $$ y_{ijt} = \beta_{0} + \beta_{1} \left( {x_{ijt} - \overline{x}_{jt} } \right) + \beta_{2} \overline{x}_{jt} + \beta_{3} z_{jt} \left( {x_{itj} - \overline{x}_{jt} } \right) + \beta_{4} z_{jt} + \delta_{t} + u_{j} + u_{jt} + e_{itj} . $$

We refer to (3) as $M_{{\overline{L1} }}$ since the level 1 individual-level variable $x_{ijt}$ is centered. This model differs from (1) because it does not center the interaction term directly, does not include the mean interaction term at the group-year level as a covariate, and does not center the group-year level affiliation status at the group-level.

Finally, one might combine the modeling framework for within- and between-effects in clustered or grouped data (reflected in (3)) with the comparative longitudinal data models that disentangle the longitudinal and cross-sectional effects of affiliation to estimate the following model:

4 $$ y_{ijt} = \beta_{0} + \beta_{1} \left( {x_{ijt} - \overline{x}_{jt} } \right) + \beta_{2} \overline{x}_{jt} + \beta_{3} z_{jt} \left( {x_{itj} - \overline{x}_{jt} } \right) + \beta_{4} \left( {z_{jt} - \overline{z}_{j} } \right) + \beta_{5} \overline{z}_{j} + \delta_{t} + u_{j} + u_{jt} + e_{itj} . $$

We refer to (4) as $M_{{\overline{L1} ,\overline{L2} }}$ since the level 1 individual-level variable $x_{ijt}$ and the level 2 group-year level variable $z_{jt}$ are both centered. This model differs from (1) because it does not center the interaction term directly or include the mean interaction term at the group-year level as a covariate.

For illustration, we also include a model that is like the one we propose (1), but excludes the terms capturing the between-PO effects, e.g.,

5 $$ y_{ijt} = \beta_{0} + \beta_{w1} \left( {x_{ijt} - \overline{x}_{jt} } \right) + \beta_{w2} \left( {I_{ijt} - \overline{I}_{jt} } \right) + \beta_{w3} \left( {z_{jt} - \overline{z}_{j} } \right) + \delta_{t} + u_{j} + u_{jt} + e_{ijt} . $$

We refer to this model as $M_{w}$.

For each of the models discussed, we have included PO and PO-year effects, $u_{j}$ and $u_{jt}$, however we also implement versions of these models that exclude the PO-year effects, and just include PO effects. Table 1 provides a tabular description of each model, indicating which terms are centered versus uncentered, included versus excluded, and what types of random (or fixed) PO or PO-year effects are included.

**Table 1:** Description of each model compared in simulations

Model											
Coefficient	$M_{wb} , L3$	$M_{wb} , L3,L2$	$M_{RE} , L3$	$M_{RE} , L3,L2$	$M_{FE}$	$M_{{\overline{L1} }} ,L3$	$M_{{\overline{L1} }} ,L3,L2$	$M_{{\overline{L1} ,\overline{L2} }} ,L3$	$M_{{\overline{L1} ,\overline{L2} }} ,L3,L2$	$M_{w} ,L3$	$M_{w} ,L3,L2$
Beneficiary dual	Centered	Centered	Not centered	Not centered	Not centered	Centered	Centered	Centered	Centered	Centered	Centered
PO affiliation	Centered	Centered	Not centered	Not centered	Not centered	Not centered	Not centered	Centered	Centered	Centered	Centered
Interaction (dual:affiliation)	Centered	Centered	Not centered	Not centered	Not centered	Dual centered	Dual centered	Dual centered	Dual centered	Centered	Centered
Percent dual (PO-year level)	Included	Included	Excluded	Excluded	Excluded	Included	Included	Included	Included	Excluded	Excluded
mean interaction (PO-year level)	Included	Included	Excluded	Excluded	Excluded	Excluded	Excluded	Excluded	Excluded	Excluded	Excluded
PO effects	Random	Random	Random	Random	Fixed	Random	Random	Random	Random	Random	Random
PO-year effects	None	Random	None	Random	None	None	Random	None	Random	None	Random

Simulation Study 1. We know that model (1) will capture the disparities of interest as defined in the Methods section. Therefore, we generate data of a similar structure to our motivating application and compare each of the models described above in terms of how closely they estimate the disparities of interest, to illustrate how much bias is obtained by using a different model than the one proposed.

We assume 1000 POs are observed over two years, and generate individuals or beneficiaries nested within PO-years. All POs are assumed unaffiliated in year 1, and in year 2, affiliation status is randomly generated for each PO with probability 0.5 of being affiliated (e.g., $z_{j1} = 0$, and $z_{j2} \sim {\mathrm{Bern}}\left( {0.5} \right)$). To create individual-level dual status flags we generate a percent dual value for each PO in each year, $p_{jt}$, with $p_{j2} \sim {\mathrm{uniform}}\left( {0.2,0.8} \right)$, and some random noise with standard deviation 0.05 added for year $t = 1$, to reflect realistic small changes in the composition of a PO over time. We then sample individual-level dual status as $x_{ijt} \sim {\mathrm{Bern}}\left( {p_{jt} } \right)$, for $i = 1, \ldots ,n_{jt}$, where $n_{jt} = 10$. We next generate PO-year level and PO-level random effects, with $\gamma_{j2} \sim N\left( {0,\sigma_{u2}^{2} } \right)$, $\gamma_{j1} \sim N\left( {0,\sigma_{u2}^{2} } \right)$, and $\gamma_{j} \sim N\left( {0,\sigma_{u1}^{2} } \right)$.

Finally, we simulate individual-level outcomes using (1), with realistic parameter values corresponding to $\beta_{0} = 50$, $\beta_{w1} = - 10$, $\beta_{b1} = - 20$, $\beta_{w2} = 1$, $\beta_{b2} = - 1$, $\beta_{w3} = 1$, $\beta_{b3} = 0$, and $\delta_{2} = 0$. This set-up corresponds to a within-PO effect of dual of $- 10$, and a between-PO effect of dual of $- 20$. In this scenario, affiliation leads to an improvement in within-PO disparities by 1 point, and a worsening of between-PO disparities by 1 point. We let $\sigma_{e} = 10$, $\sigma_{u1} = 10$, and $\sigma_{u2} = 5$, so there is within-PO and between-year variation not fully accounted for by the model, though it is smaller than the PO-level variation.

We repeat the process of simulating data 500 times to obtain 500 simulated datasets for analysis, and for each dataset, we fit our proposed model, $M_{wb}$, and the other comparison models, $M_{FE}$, $M_{RE}$, $M_{{\overline{L1} }}$, $M_{{\overline{L1} ,\overline{L2} }}$, and $M_{w}$. We calculate the bias for each quantity of interest under each model (difference between the estimated and true parameter value) and compare across models using absolute bias and a relative bias measure, where bias is expressed relative to the standard deviation of the simulated outcomes.

Tables 2 and 3 display the bias and relative bias for each parameter of interest from each model. The models that center the individual-level dual status ($M_{wb}$,$M_{{\overline{L1} }}$, $M_{{\overline{L1} ,\overline{L2} }}$, $M_{w}$) have the least bias for within-PO disparities, and the simple random effects models without centering, $M_{RE}$, have the most bias. This is the same pattern observed for the effect of affiliation on within-PO disparities. For between-PO disparities, the proposed model $M_{wb}$ has the least bias, and it is the only one that estimates the effect of affiliation on between-PO disparities. For the main effect of affiliation, which is the effect of affiliation on quality within POs for non-duals (in POs without any duals), $M_{wb}$ also has the least bias. There is not a meaningful difference between the version of a model with both PO-year and PO random effects versus one with just PO random effects, though the one with PO and PO-year random effects has slightly less bias.

**Table 2:** Bias for within-PO disparities, between-PO disparities, effect of affiliation on within-PO disparities, effect of affiliation on between-PO disparities, and effect of affiliation on non-duals from Simulation 1

Model											
parameter	$M_{wb} , L3$	$M_{wb} , L3,L2$	$M_{RE} , L3$	$M_{RE} , L3,L2$	$M_{FE}$	$M_{{\overline{L1} }} ,L3$	$M_{{\overline{L1} }} ,L3,L2$	$M_{{\overline{L1} ,\overline{L2} }} ,L3$	$M_{{\overline{L1} ,\overline{L2} }} ,L3,L2$	$M_{w} ,L3$	$M_{w} ,L3,L2$
Within-PO Disparities	− 0.014	− 0.014	− 0.550	− 0.640	− 0.247	− 0.014	− 0.014	− 0.014	− 0.014	− 0.014	− 0.014
Between-PO Disparities	− 0.155	− 0.130				− 0.343	− 0.339	− 0.343	− 0.337		
Affiliation effect on Within-PO disparities	0.020	0.020	− 0.190	− 0.201	− 0.053	0.020	0.020	0.020	0.020	0.020	0.020
Affiliation effect on Between- PO disparities	0.194	0.116									
Affiliation effect for Non-duals	− 0.155	− 0.117	− 0.963	− 1.001	− 1.162	− 0.601	− 0.680	− 0.559	− 0.559	− 0.558	− 0.558

Blank cells indicate the model does not estimate the parameter. The models are as follows: $$M_{wb}$$ is the proposed model with centered fixed effects for dual status, affiliation status, and their interaction, as well as PO-year level percent dual, the PO-year level mean interaction term and the mean affiliation status for each PO. L3 indicates random effects at the PO level, and L3, L2 indicates random effects at the PO-year and PO level. $$M_{RE}$$ includes dual status, affiliation status, and their interaction as fixed effects (with no centering). $$M_{FE}$$ is like $$M_{RE}$$ but uses fixed effects for PO rather than random effects. $$M_{{\overline{L1} }}$$ is like $$M_{wb} $$ but does not center affiliation status (or include PO-level mean affiliation status or the PO-year level mean interaction term as a covariate). $$M_{{\overline{L1} ,\overline{L2} }}$$ is like $$M_{{_{{\overline{L1} }} }}$$ except it also centers affiliation status and includes the mean affiliation status for each PO. $$M_{w}$$ centers all terms in the same way as $$M_{wb}$$, but does not include the PO-year level percent dual, or PO-year level mean interaction term, or PO-level mean affiliation status as additional covariates. All models include year dummy variables

**Table 3:** Relative bias for within-PO disparities, between-PO disparities, effect of affiliation on within-PO disparities, effect of affiliation on between-PO disparities, and effect of affiliation on non-duals from Simulation 1

Model											
Parameter	$M_{wb} , L3$	$M_{wb} , L3,L2$	$M_{RE} , L3$	$M_{RE} , L3,L2$	$M_{FE}$	$M_{{\overline{L1} }} ,L3$	$M_{{\overline{L1} }} ,L3,L2$	$M_{{\overline{L1} ,\overline{L2} }} ,L3$	$M_{{\overline{L1} ,\overline{L2} }} ,L3,L2$	$M_{w} ,L3$	$M_{w} ,L3,L2$
Within-PO Disparities	− 0.001	− 0.001	− 0.034	− 0.039	− 0.015	− 0.001	− 0.001	− 0.001	− 0.001	− 0.001	− 0.001
Between-PO Disparities	− 0.010	− 0.008				− 0.021	− 0.021	− 0.021	− 0.021		
Affiliation effect on Within-PO disparities	0.001	0.001	− 0.012	− 0.012	− 0.003	0.001	0.001	0.001	0.001	0.001	0.001
Affiliation effect on Between- PO disparities	0.012	0.007									
Affiliation effect for Non-duals	− 0.010	− 0.007	− 0.059	− 0.062	− 0.071	− 0.037	− 0.042	− 0.034	− 0.034	− 0.034	− 0.034

Relative bias is bias divided by the standard deviation of the outcomes. Blank cells indicate the model does not estimate the parameter*.* The models are as follows: $M_{wb}$ is the proposed model with centered fixed effects for dual status, affiliation status, and their interaction, as well as PO-year level percent dual, the PO-year level mean interaction term and the mean affiliation status for each PO. L3 indicates random effects at the PO level, and L3, L2 indicates random effects at the PO-year and PO level. $M_{RE}$ includes dual status, affiliation status, and their interaction as fixed effects (with no centering). $M_{FE}$ is like $M_{RE}$ but uses fixed effects for PO rather than random effects. $M_{{\overline{L1} }}$ is like $M_{wb} { }$ but does not center affiliation status (or include PO-level mean affiliation status or the PO-year level mean interaction term as a covariate). $M_{{\overline{L1} ,\overline{L2} }}$ is like $M_{{\overline{L1} }}$ except it also centers affiliation status and includes the mean affiliation status for each PO. $M_{w}$ centers all terms in the same way as $M_{wb}$, but does not include the PO-year level percent dual, or PO-year level mean interaction term, or PO-level mean affiliation status as additional covariates. All models include year dummy variables.

The takeaway from this simulation is that to estimate all disparities and affiliation effects of interest, the proposed model $M_{wb}$ is necessary. Additionally, all parameters estimated by $M_{RE}$ and $M_{FE}$ have more bias than those estimated by $M_{wb}$, two of four of the parameters estimated by $M_{{\overline{L1} }}$ and $M_{{\overline{L1} ,\overline{L2} }}$ have more bias, and $M_{w}$ produces more bias for the main effect of affiliation.

Simulation Study 2. Next, we simulate comparative longitudinal data similar in structure to our motivating application, but not exactly from the proposed model. As with Simulation 1, all POs are assumed unaffiliated in year 1, and we generate affiliation status in year 2, and PO-year-level percent dual, and individual-level dual status in the same way. We next generate PO-year level quality effects as $\gamma_{j1} \sim N\left( {50 - 20\overline{x}_{j1} ,\sigma_{u1}^{2} } \right)$ and $\gamma_{j2} \sim N\left( {50 - 20\overline{x}_{j2} - 5z_{j2} \overline{x}_{j2} ,\sigma_{u1}^{2} } \right)$, which encodes a negative contextual and between-PO effect of dual eligibility on quality, and a negative effect of affiliation on contextual effects and between-PO disparities. We additionally generate random PO effects, encoding the belief that POs have different levels of quality that persist over time that aren’t explained by the composition of the PO, with $\gamma_{j} \sim N\left( {0,\sigma_{u1}^{2} } \right)$. Finally, we simulate individual-level outcomes as follows: $y_{ijt} \sim N\left( {\gamma_{jt} + \gamma_{j} - 10x_{ijt} + z_{jt} + x_{ijt} z_{jt} ,\sigma_{e}^{2} } \right)$, for $j = 1, \ldots ,100$, $t = 1,2$ and $i = 1,2, \ldots ,10$.

This set-up corresponds to a within-PO effect of dual-eligibility of $- 10$, a contextual effect of $- 20$, and thus a between-PO effect of dual of $- 30$. In this scenario, affiliation leads to an improvement in quality for non-duals of 1 point, but it also reduces disparities within POs by 1 point. However, affiliation leads to between-PO disparities that are larger (worsened) by 4 points (after affiliation, within-PO disparities are reduced by 1 point and contextual effects are of dual worsened by 5 points, leading to a worsening of between-PO disparities by 4 points). We let $\sigma_{e} = 10$, $\sigma_{u1} = 10$, and $\sigma_{u2} = 5$, as in Simulation 1, and conduct repeated simulations to compare bias for each quantity of interest under each model.

Table 4 displays the relative bias for each parameter of interest from each model. Consistently with the prior simulation scenario, the models that center the individual-level dual status ($M_{wb}$,$M_{{\overline{L1} }}$,$M_{{\overline{L1} ,\overline{L2} }}$, $M_{w}$) have by far the least bias for within-PO disparities, and much less than the random effects models without centering. This is the same pattern observed for the effect of affiliation on within-PO disparities. For between-PO disparities, the proposed model $M_{wb}$ also has the least bias by far, with PO-year and PO random effects giving slightly less bias than just PO random effects. For the main effect of affiliation, $M_{wb}$ also has the least bias, however PO random effects only gives less bias than PO-year and PO random effects.

**Table 4:** Relative bias for within-PO disparities, between-PO disparities, effect of affiliation on within-PO disparities, effect of affiliation on between-PO disparities, and effect of affiliation on non-duals from Simulation 2

Model											
parameter	$M_{wb} , L3$	$M_{wb} , L3,L2$	$M_{RE} , L3$	$M_{RE} , L3,L2$	$M_{FE}$	$M_{{\overline{L1} }} ,L3$	$M_{{\overline{L1} }} ,L3,L2$	$M_{{\overline{L1} ,\overline{L2} }} ,L3$	$M_{{\overline{L1} ,\overline{L2} }} ,L3,L2$	$M_{w} ,L3$	$M_{w} ,L3,L2$
Within-PO Disparities	< 0.001	< 0.001	− 0.059	− 0.069	− 0.022	< 0.001	< 0.001	< 0.001	< 0.001	< 0.001	< 0.001
Between-PO Disparities	− 0.002	− 0.001				− 0.056	− 0.056	− 0.056	− 0.056		
Affiliation effect on Within-PO disparities	< 0.001	< 0.001	− 0.035	− 0.036	− 0.009	< 0.001	< 0.001	< 0.001	< 0.001	< 0.001	< 0.001
Affiliation effect on Between- PO disparities	< 0.001	0.003									
Affiliation effect for non-duals	< 0.001	− 0.001	− 0.127	− 0.126	− 0.140	− 0.115	− 0.115	− 0.114	− 0.114	− 0.116	− 0.116

Relative bias is bias divided by the standard deviation of the outcomes. Blank cells indicate the model does not estimate the parameter. The models are as follows: $M_{wb}$ is the proposed model with centered fixed effects for dual status, affiliation status, and their interaction, as well as PO-year level percent dual, the PO-year level mean interaction term and the mean affiliation status for each PO. L3 indicates random effects at the PO level, and L3, L2 indicates random effects at the PO-year and PO level. $M_{RE}$ includes dual status, affiliation status, and their interaction as fixed effects (with no centering). $M_{FE}$ is like $M_{RE}$ but uses fixed effects for PO rather than random effects. $M_{{\overline{L1} }}$ is like $M_{wb} { }$ but does not center affiliation status (or include PO-level mean affiliation status or the PO-year level mean interaction term as a covariate). $M_{{\overline{L1} ,\overline{L2} }}$ is like $M_{{\overline{L1} }}$ except it also centers affiliation status and includes the mean affiliation status for each PO. $M_{w}$ centers all terms in the same way as $M_{wb}$, but does not include the PO-year level percent dual, or PO-year level mean interaction term, or PO-level mean affiliation status as additional covariates. All models include year dummy variables

The overall conclusion from these simulations is that the proposed model $M_{wb}$ provides estimates for all quantities of interest and has the least bias. The choice to use PO versus PO and PO-year random effects should be based on the data, and whether the results indicate PO-year random effects are needed in addition to PO random effects.

### The effect of health system affiliation on disparities in diabetes medication adherence

We now apply the proposed model, $M_{wb}$, to estimate the effects of affiliation on within- and between-PO disparities in the diabetes medication adherence measure, a quality measure for evaluating the performance of healthcare providers, and defined as the percent of members with a prescription for diabetes medications who fill their prescription often enough to cover 80% or more of the time they are supposed to be taking the medication. Levin et al. found some evidence of worsening disparities in medication adherence rates following health system affiliation of PCPs (Levin et al. 2023). Our methods focus on disparities within POs as meaningful units, since most strategies to address disparities are at the organization-level (data infrastructure, provider training, quality improvement processes, etc.), and the decomposition analysis provides a deeper understanding of equity.

**Fig. 1:**
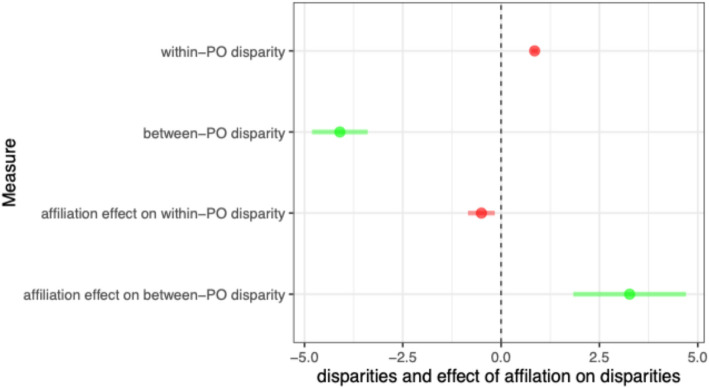
Estimated within- and between-PO disparities and effect of affiliation on within- and between-PO disparities for diabetes medication adherence measure

$M_{wb}$ was applied with PO and PO-year random effects, and with adjustment for standard beneficiary and PO characteristics described in Methods. Figure 1 plots the point estimates and 95% confidence intervals for within-PO and between-PO disparities in unaffiliated POs ($\beta_{w1}$ and $\beta_{b1} )$, which were positive and negative respectively, indicating that in unaffiliated POs, duals were actually doing better on this measure within POs, but were concentrated in poorer performing POs. However, affiliation had a negative effect on within-PO disparities (captured by $\beta_{w3}$), meaning quality for duals got worse relative to non-duals compared to pre-affiliation quality levels, but a positive effect on between-PO disparities (captured by $\beta_{b3} )$ indicating that there are smaller differences in the quality of affiliated POs that serve duals vs. those that serve non-duals.

## Discussion

Health system affiliated practices are expanding rapidly across the U.S., but the impacts on socioeconomic disparities are not clear. System affiliation may impact disparities within POs, such that becoming affiliated leads to improved or worsened disparities within that PO. While it is well known that socioeconomically disadvantaged individuals tend to be concentrated within poorer performing providers, the effect of affiliation on between-provider disparities is unknown.

This paper provides statistical models that can be used to estimate the effect of an intervention (health system affiliation) on within- and between-group disparities. While we focused here on dual vs. non-dual disparities, these methods are applicable to other types of disparities including racial/ethnic disparities, and other measures of socioeconomic status. They can be applied to other settings where an intervention is applied at the group level and expected to impact disparities, and thus gaining an understanding of the impact on disparities both within- and between-groups is informative for policy and decision-making regarding the utility of the intervention.

Although this model can be used to provide a detailed understanding of disparities and the impact of an intervention on disparities by capturing both baseline within- and between-group disparities and the impact of the intervention on these disparities, this also means that this model requires sufficiently rich data to be able to estimate all regression coefficients. With large data sets like those used in this application, there also needs to be sufficient variation in the composition of POs (e.g., PO-level percent dual) before and after affiliation, and the number of POs that become affiliated needs to be large enough to estimate effects of affiliation on disparities.

These methods make similar assumptions to traditional difference-in-difference models, for example that, conditional on other covariates, trends in within-PO disparities would have evolved in parallel for affiliating POs and independent POs, in the absence of affiliation. It is therefore useful to control for a rich set of covariates thought to be associated with the evolution of the outcomes to strengthen the premise for conditional parallel trends. In our case, we controlled for characteristics of patients and POs, as these are associated with affiliation status and quality of care outcomes. To further increase robustness to selection bias and compositional changes we used a balanced panel of POs. If feasible, a balanced panel of beneficiaries could also be used. Finally, propensity score weights could also be included in the regression model, to balance the affiliated and independent POs on observable characteristics.

Future work will consider how to expand these models to allow for effects of affiliation to differ by affiliation cohort and years since becoming affiliated, in the spirit of the large body of literature in economics developing differences in differences models that allow for heterogeneity in treatment effects by cohort and time (Callaway and Sant’Anna 2021; Roth et al. 2023).

## Data Availability

The data that support the findings of this study are available from CMS. Restrictions apply to the availability of these data, which were used under a data use agreement for this study.

## Appendix

While not of primary interest in this application, we may also want to know the effect of affiliation on quality for duals and non-duals, $\alpha_{d}$, and $\alpha_{n}$. The effect of affiliation on quality for non-duals is the difference in expected quality for a non-dual post- and pre-affiliation, in a PO with $\overline{X}_{jt} = \overline{x}_{jt}$ and $\overline{Z}_{j} = \overline{z}_{j}$. This can be defined as

$$ \begin{aligned} \alpha_{n} = & E\left( {Y_{ijt} |X_{ijt} = 0,\overline{X}_{jt} = \overline{x}_{jt} ,Z_{jt} = 1,\overline{Z}_{j} = \overline{z}_{j} } \right) - E\left( {Y_{ijt} |X_{ijt} = 0,\overline{X}_{jt} = \overline{x}_{jt} ,Z_{jt} = 0,\overline{Z}_{j} = \overline{z}_{j} } \right) \\ = & (\beta_{0} + \beta_{w1} \left( { - \overline{x}_{jt} } \right) + \beta_{b1} \overline{x}_{jt} + \beta_{b2} \overline{x}_{jt} + \beta_{w2} \left( { - \overline{x}_{jt} } \right) + \beta_{w3} \left( {1 - \overline{z}_{j} } \right) + \beta_{b3} \overline{z}_{j} ) \\ & - \left( {\beta_{0} + \beta_{w1} \left( { - \overline{x}_{jt} } \right) + \beta_{b1} \overline{x}_{jt} + \beta_{w3} \left( { - \overline{z}_{j} } \right) + \beta_{b3} \overline{z}_{j} } \right) \\ = & \left( {\beta_{b2} - \beta_{w2} } \right)\overline{x}_{jt} + \beta_{w3} . \\ \end{aligned} $$

Similarly, the effect of affiliation on quality for duals is the difference in expected quality for a dual post- and pre-affiliation. This can be defined as

$$ \begin{aligned} \alpha_{d} = & E\left( {Y_{ijt} |X_{ijt} = 1,\overline{X}_{jt} = \overline{x}_{jt} ,Z_{jt} = 1,\overline{Z}_{j} = \overline{z}_{j} } \right) - E\left( {Y_{ijt} |X_{ijt} = 1,\overline{X}_{jt} = \overline{x}_{jt} ,Z_{jt} = 0,\overline{Z}_{j} = \overline{z}_{j} } \right) \\ = & \beta_{0} + \beta_{w1} \left( {1 - \overline{x}_{jt} } \right) + \beta_{b1} \overline{x}_{jt} + \beta_{b2} \overline{x}_{jt} + \beta_{w2} \left( {1 - \overline{x}_{jt} } \right) + \beta_{w3} \left( {1 - \overline{z}_{j} } \right) \\ & + \beta_{b3} \overline{z}_{j} - \left( {\beta_{0} + \beta_{w1} \left( {1 - \overline{x}_{jt} } \right) + \beta_{b1} \overline{x}_{jt} + \beta_{w3} \left( { - \overline{z}_{j} } \right) + \beta_{b3} \overline{z}_{j} } \right) \\ = & \left( {\beta_{b2} - \beta_{w2} } \right)\overline{x}_{jt} + \beta_{w2} + \beta_{w3} . \\ \end{aligned} $$
